# Examining the Effects of (α4)3(β2)2 Nicotinic Acetylcholine Receptor-Selective Positive Allosteric Modulator on Acute Thermal Nociception in Rats

**DOI:** 10.3390/molecules25122923

**Published:** 2020-06-25

**Authors:** Farah Deba, Kara Ramos, Matthew Vannoy, Kemburli Munoz, Lois S. Akinola, M. Imad Damaj, Ayman K. Hamouda

**Affiliations:** 1 Department of Pharmaceutical Sciences, College of Pharmacy, The University of Texas at Tyler, Tyler, TX 75799, USA; Fdeba@uttyler.edu; 2 Department of Pharmaceutical Sciences, College of Pharmacy, Texas A&M Health Sciences Center MS 131, 1010 W. Ave. B, Kingsville, TX 78363, USA; ramoskara09@yahoo.com (K.R.); matthewvannoy19@gmail.com (M.V.); munozkim0903@gmail.com (K.M.); 3 Department of Pharmacology and Toxicology, Medical College of Virginia, Virginia Commonwealth University, Kontos Medical Science Building, 1217 E. Marshall St., P.O. Box 980613, Richmond, VA 23298, USA; akinolals@vcu.edu (L.S.A.); m.damaj@vcuhealth.org (M.I.D.)

**Keywords:** nicotinic acetylcholine receptors (nAChRs), positive allosteric modulators (PAMs), desformylflustrabromine (dFBr), CMPI, acute thermal nociception, hot plate, tail flick

## Abstract

Neuronal nicotinic acetylcholine receptor (nAChR)-based therapeutics are sought as a potential alternative strategy to opioids for pain management. In this study, we examine the antinociceptive effects of 3-(2-chlorophenyl)-5-(5-methyl-1-(piperidin-4-yl)-1*H*-pyrazol-4-yl)isoxazole (CMPI), a novel positive allosteric modulator (PAM), with preferential selectivity to the low agonist sensitivity (α4)3(β2)2 nAChR and desformylflustrabromine (dFBr), a PAM for α4-containing nAChRs. We used hot plate and tail flick tests to measure the effect of dFBr and CMPI on the latency to acute thermal nociceptive responses in rats. Intraperitoneal injection of dFBr, but not CMPI, dose-dependently increased latency in the hot plate test. In the tail flick test, the effect achieved at the highest dFBr or CMPI dose tested was only <20% of the maximum possible effects reported for nicotine and other nicotinic agonists. Moreover, the coadministration of dFBr did not enhance the antinociceptive effect of a low dose of nicotine. Our results show that the direct acute effect of dFBr is superior to that for CMPI, indicating that selectivity to (α4)3(β2)2 nAChR is not advantageous in alleviating responses to acute thermal nociceptive stimulus. However, further studies are necessary to test the suitability of (α4)3(β2)2 nAChR-selective PAMs in chronic pain models.

## 1. Introduction

Neuronal nicotinic acetylcholine receptors (nAChRs) containing the α4 and β2 nAChR subunits (herein referred to as α4β2* nAChRs with asterisks, to indicate the diversity of subunit composition) are the most predominant nAChR subtypes in the brain [1,2,3]. The α4β2* nAChRs are expressed in neuronal circuitries involved in the transmission, perception, and modulation of painful stimuli and contribute to the underlying pathophysiological processes of neuropathic and inflammatory pain [4,5,6]. Compounds targeting the α4β2* nAChRs hold promise in the development of non-opioid analgesics [6,7,8,9]. The antinociceptive efficacy of α4β2* nAChRs agonists has been demonstrated in animal models, including those for acute thermal pain, neuropathic pain, pain associated with inflammatory conditions, and chemotherapy-induced painful neuropathy [10,11,12,13]. The development of clinically relevant nicotinic agonists was proven difficult, despite their potent antinociceptive and anti-inflammatory effects in a variety of preclinical models. Doses of nAChR agonists that produced analgesic effects also produced side effects, due to interactions at other nAChRs subtypes (e.g., α3-containing nAChRs) [14,15]. Therefore, the positive allosteric modulator (PAM) of nAChRs emerged as a class of therapeutic agents to overcome the pharmacological selectivity and safety issues associated with agonists [16,17,18,19,20,21].

By definition, PAMs of nAChR bind at allosteric sites other than the agonist binding sites. Therefore, they do not replace the agonist nor directly activate the nAChR channel on their own. Instead, they bind simultaneously with an agonist, increasing its potency, efficacy, or both, which results in the enhancement of nAChR function [22]. As such, PAMs avoid two unwanted pharmacological properties seen with nAChR agonists: 1) binding at ACh binding sites which are highly conserved among multiple nAChR subtypes, resulting in unwanted side effects [8]; and 2) changes in nAChRs dynamics (e.g., continuous activation and desensitization) which can result in unwanted effects on the pattern of cholinergic neuronal activities [16]. In in vitro studies, many nAChR PAMs have exhibited a higher degree of nAChR subtype selectivity than agonists [23,24,25]. In in vivo studies, PAMs of nAChRs have antinociceptive and anti-inflammatory effects of their own and improve the analgesic effects, without increasing side effects when coadministered with α4β2 nAChR agonists [4,15,26,27,28].

**Figure 1:**
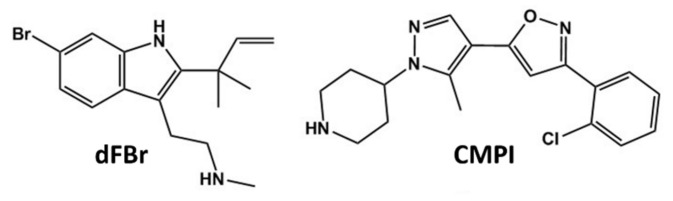
Structures of α4β2* neuronal nicotinic acetylcholine receptors (nAChRs) positive allosteric modulators (PAMs), desformylflustrabromine (dFBr) and 3-(2-chlorophenyl)-5-(5-methyl-1-(piperidin-4-yl)-1*H*-pyrazol-4-yl)isoxazole (CMPI).

The two α4β2* nAChR isoforms, the low agonist sensitivity isoform ((α4)3(β2)2) nAChR comprised of three alpha and two beta subunits) and the high agonist sensitivity isoform ((α4)2(β2)3 nAChR comprised of two alpha and three beta subunits), differ in their biochemical and pharmacological characteristics and potentially differ in their in vivo expression patterns and their roles in different central nervous system functions [3,29,30]. Therefore, we reasoned that nAChR PAM subtype selectivity ((α4)3(β2)2 vs. (α4)2(β2)3 nAChR) would translate to differences in the in vivo efficacy. In this study, we examine the antinociceptive effects of 3-(2-chlorophenyl)-5-(5-methyl-1-(piperidin-4-yl)-1*H*-pyrazol-4-yl)isoxazole (CMPI) [24], a PAM with a unique selectivity to the low agonist sensitivity (α4)3(β2)2 nAChR [24,31,32] and the results were compared with those obtained with dFBr (desformylflustrabromine, N-(2-[6-bromo-2(1,1-dimethyl-2-propyl)-1H-indol-3-yl]ethyl-N-methylamine), a PAM of α4-containing nAChRs (Figure 1) [23,33,34]. We used hot plate and tail flick tests for acute thermal nociception as two well-established paradigms in which the α4β2* nAChRs play an essential role. Our results show that the direct acute effect of CMPI on latency to acute thermal stimuli is minimal, and not comparable to that seen with non-selective nAChR agonists, like nicotine, or with PAMs with broader nAChR subtype coverage, like dFBr. These results indicate that selectivity to (α4)3(β2)2 nAChR may not be advantageous in treating acute pain conditions. However, these results do not preclude their potential suitability for the treatment of chronic pain conditions, and further studies are warranted to test the antinociceptive and anti-allodynic effect of CMPI in models for neuropathic and inflammatory pain.

## 2. Results

### 2.1. Effect of dFBr and CMPI on Acute Thermal Nociception Behavior in Hot Plate Test

**Figure 2:**
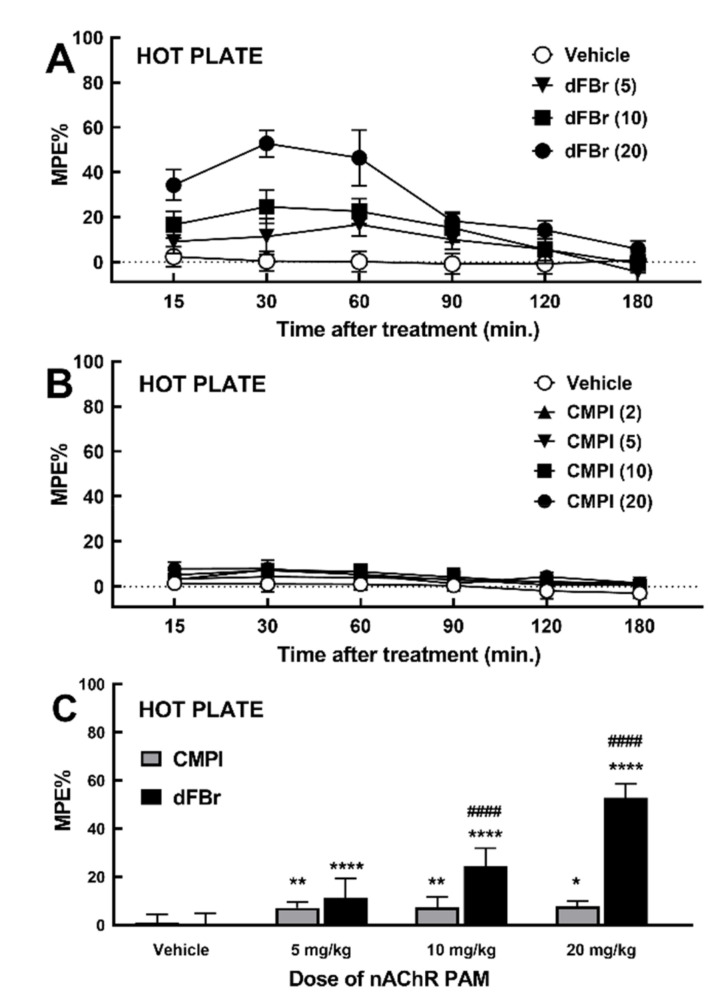
(**A** and **B**) Time course of the effects of dFBr and CMPI, respectively, on acute thermal nociception in the hot plate test. Rats received i.p. injection of vehicle or increasing dose (in mg/kg) of dFBr or CMPI, then subjected to the hot plate at the time indicated. Data are shown as mean % MPE ± SD of at least six rats per each treatment group, as detailed under Materials and Methods (Section 4.3.1). (**C**) Peak dFBr and CMPI effect at 30 min post-injection were analyzed using two-way ANOVA with Holm–Sidak’s multiple comparisons test. *p < 0.05, **p < 0.01, ***p < 0.001, ****p <0.0001 indicates statistical significance between drug treatment versus vehicle group. ^####^*p* <0.0001 indicates the significant difference between dFBr treatment versus treatment with the same dose of CMPI.

The acute thermal pain latency before and after intraperitoneal (i.p.) injection of vehicle (a mixture of 90% saline, 5% Tween 80, and 5% propylene glycol), dFBr (5, 10, or 20 mg/kg), or CMPI (2, 5, 10, 15, or 20 mg/kg) on Sprague–Dawley rats in the hot plate test was determined using a Hot Plate Analgesia Meter (IITC Inc.) maintained at 55 °C. Figure 2A,B show the time course of dFBr and CMPI effects, respectively, on acute pain latency in the hot plate test, expressed as a percentage of the maximum possible effect (MPE %). Following i.p. injection, dFBr dose-dependently increased the latency (time for hind paw-licking behavior) in the hot plate test. A fast onset characterized the dFBr effect; effect starts within 15 min, and the maximum effect was achieved within 30 min posttreatment. The time course of dFBr dose-response was statistically significant in two-way ANOVA (F_dFBr dosexTime_ (15, 228) = 24.17, *p* < 0.0001). The effect of dFBr was reversible, and the hot plate thermal latenare shown cy returned to within ± 5% of pretreatment baseline latency after 180 min. In contrast, the effect of CMPI on hot plate thermal latency was less than 10% of MPE at all CMPI doses tested (Figure 1B). The time course of CMPI dose-response was much less significant in two-way ANOVA (F_CMPI dosexTime_ (20, 348) = 1.737, p = 0.0264) than dFBr. At 30 min following treatment, dFBr at 5, 10, and 20 mg/kg produced a statistically significant effect (*p* < 0.0001) when compared with vehicle using Holm–Sidak’s multiple comparison test (Figure 2C). The effect of dFBr at 10 and 20 mg/kg was statistically significant (*p* < 0.0001) compared with pretreatment with an equivalent dose of CMPI. CMPI at 5, 10, and 20 mg/kg was also significant when compared with the vehicle with *p* values of 0.0041, 0.0026, and 0.0711, respectively.

### 2.2. Effect of dFBr and CMPI on Acute Thermal Nociception Behavior in Tail Flick Test

**Figure 3:**
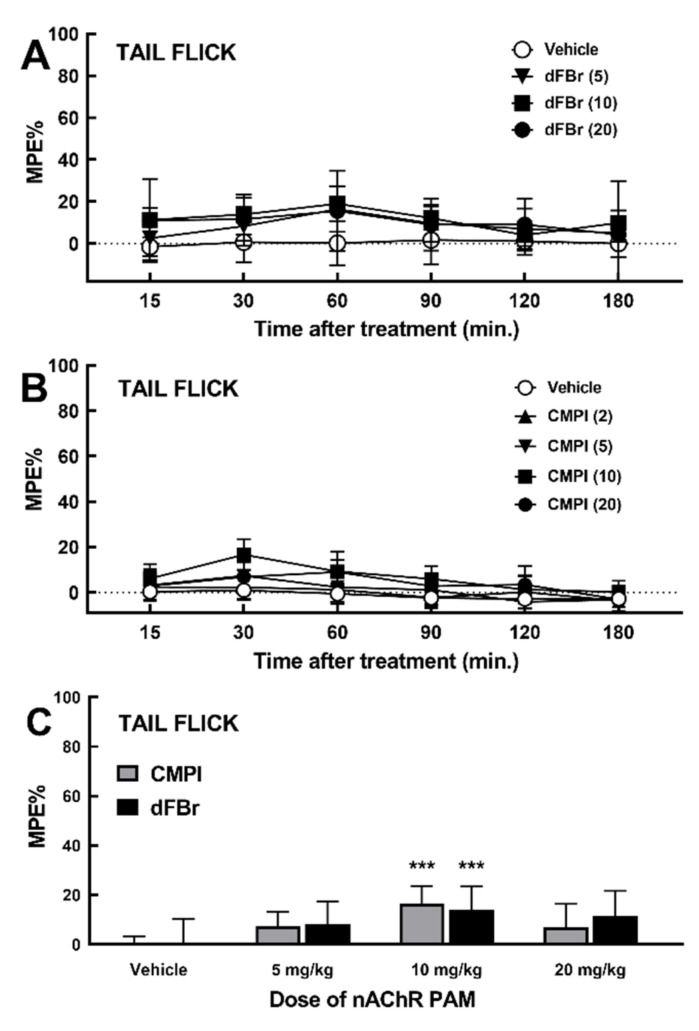
(**A** and **B**) Time course of the effects of increasing dose of dFBr and CMPI, respectively, on acute thermal nociception in the tail flick test. Rats were pretreated with i.p. injection of vehicle, dFBr (5, 10, or 20 mg/kg) or CMPI (5, 10, or 20 mg/kg), then subjected to the tail flick after 15, 30, 60, 90, 120, and 180 min. Data are shown as mean %MPE±SD of at least six rats per each treatment group, as detailed under Materials and Methods (Section 4.3.2). (**C**) Peak dFBr and CMPI effect at 30 min post-injection were analyzed using two-way ANOVA with Holm-Sidak’s multiple comparisons test. *** *p* <0.001 indicates a significant difference between drug treatment versus vehicle.

The time course of the acute thermal pain latency of Sprague–Dawley rats in tail flick measured following the i.p. injection of the vehicle, dFBr (5, 10, or 20 mg/kg), or CMPI (2, 5, 10, 15, or 20 mg/kg) are shown in Figure 3A,B. The effect of dFBr and CMPI on tail flick thermal latency was less than 20% of MPE at all CMPI and dFBr doses tested. Statistical analyses of dose-dependent effect of dFBr and CMPI at 30 min following treatment using two-way ANOVA with Holm–Sidak’s multiple comparison test (Figure 3C) revealed a statistically significant (p<0.001) difference between pretreatment with 10 mg/kg of dFBr or CMPI and vehicle pretreatment. There was no statistical significance (p >0.05) between dFBr treatments versus treatment with equivalent doses of CMPI.

### 2.3. Effect of dFBr on Nicotine-Induced Acute Thermal Antinociception

**Figure 4:**
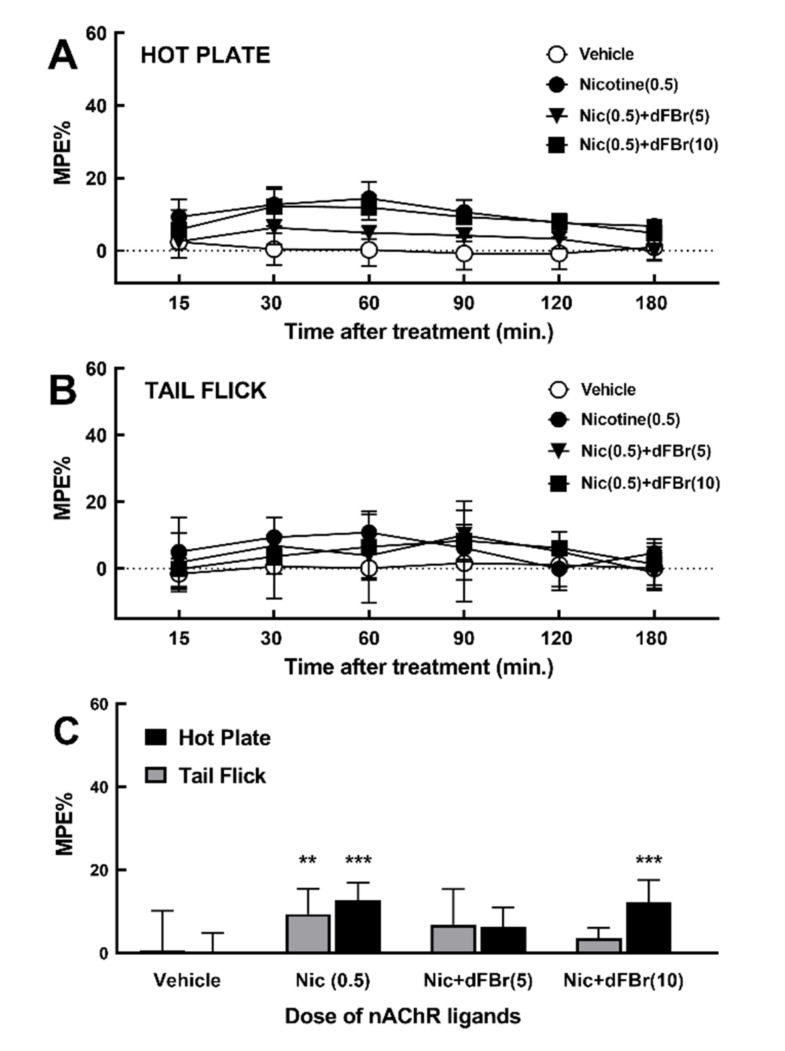
(**A** and **B**) Time course of the effects of dFBr on nicotine-induced acute thermal antinociception in the hot plate and tail flick tests, respectively. Rats were pretreated with i.p. injection of vehicle or dFBr then with s.c. injection of 0.5 mg/kg nicotine, then subjected to the hot plate or tail flick at 15, 30, 60, 90, 120, and 180 min. Data are shown as mean %MPE ± SD of at least six rats per each treatment group, as detailed under Materials and Methods (Section 4.3.2). (**C**) Peak effect at 30 min post-injection were analyzed using two-way ANOVA with Holm–Sidak’s multiple comparisons test. ** *p* < 0.01 and *** *p* < 0.001 indicate significant difference between drug treatment versus vehicle group. dFBr treatments versus treatment with nicotine alone were not statistically significant (*p* > 0.05).

To examine the effect of dFBr in combination with a low dose of nicotine on acute thermal nociception, rats were treated with nicotine (0.5 mg/kg, s.c.), with or without treatment with dFBr (5 or 10 mg/kg, i.p. injection), then subjected to the hot plate and tail flick tests (Figure 4).

Pretreatment with nicotine at 0.5 mg/kg enhanced acute thermal pain latency with %MPE of 13 ± 2 and 10 ± 2 %, in hot plate and tail flick tests. These values are consistent with previously reported values for low doses of nicotine [35]. The coadministration of dFBr with nicotine did not enhance the nicotine-induced acute thermal antinociceptive effect in the hot plate or tail flick tests. Instead, the coadministration of dFBr produced a dose-dependent decrease in nicotine-induced antinociceptive effect on tail flick test, and at 5 mg/kg, reduced the nicotine-induced antinociceptive effect in the hot plate test. Analyses of these results using two-way ANOVA with Holm–Sidak’s multiple comparison test (Figure 4C) revealed that the effect of nicotine alone was statistically significant from vehicle pretreatment (*p* < 0.001 and *p* < 0.01 in hot plate and tail flick, respectively). There were no statistically significant differences between the effect of nicotine+dFBr treatments when compared to nicotine pretreatment.

### 2.4. Rat Sex Does Not Influence dFBr and CMPI Modulation of Acute Thermal Nociception

**Figure 5:**
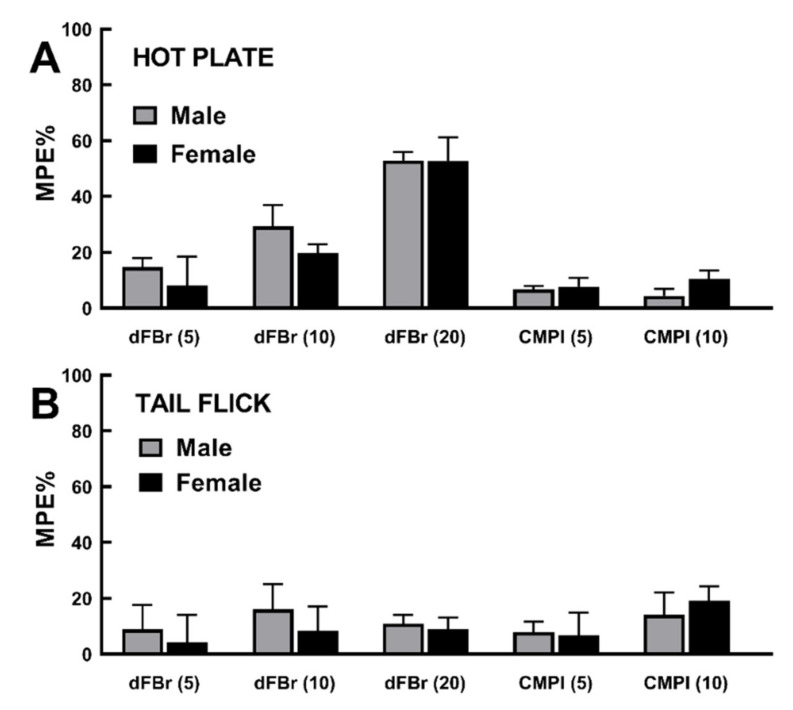
(**A**) Effect of rat sex on latency (%MPE + SD) to acute thermal nociception in the Hot Plate test (experiments in Figure 1), recorded 30 min following i.p. injection of dFBr or CMPI (dose indicated in mg/kg rat weight). (**B**) Effect of rat sex on latency (%MPE + SD) to acute thermal nociception in the Tail Flick test (experiments in Figure 2) recorded 30 min following i.p. injection of dFBr or CMPI. Data are non-significant (*p* > 0.05) based on two-way ANOVA analyses with Holm–Sidak’s multiple comparison test.

To investigate if there was a sex difference in dFBr or CMPI effects in acute thermal nociception tests, %MPE values were calculated separately for males and females within treatment groups (Figure 5), based on latency recorded in hot plate and tail flick 30 min following the i.p. injection of dFBr (5, 10, or 20 mg/kg) or CMPI (5 or 10 mg/kg) (experiments shown in Figure 1 and Figure 2). Two-way ANOVA analyses comparing male versus female to same-drug treatment response revealed no significant difference (*p* > 0.05) due to animal sex.

## 3. Discussion

The role of neuronal nAChRs as possible targets for modulation of nociception and the pathophysiology of chronic pain has been established in many animal and human studies [4,6,8]. The ability of α4β2* nAChR agonists, including epibatidine, nicotine, A-85380, and ABT-594, to reduce nociceptive responses has been demonstrated in a variety of rodent pain models [6,9]. Furthermore, PAMs of α4β2* nAChR reduced nociceptive responses in animal models of neuropathic and chemically-induced pain [27,28]. In this study, we investigated the effects of two nAChR PAMs, dFBr and CMPI, in acute thermal pain tests. dFBr is a tryptophan-derived metabolite isolated from the North Sea bryozoan *Flustra foliacea* and found to potentiate ACh-induced responses of α4β2* nAChRs [23,33]. CMPI is a piperidine derivative that has been identified via chemical synthesis and high throughput screening as a nAChR PAM [24] then found to selectively potentiate ACh-induced responses of the low agonist sensitivity (α4)3(β2)2 nAChR, but not the high agonist sensitivity (α4)2(β2)3 nAChR [31,32]. While studies to evaluate the in vivo effects of CMPI are still in their infancy, the pharmacological effects of dFBr have been examined in rodent models for nicotine self-administration, nicotine withdrawal, nicotine discriminative stimulus, neuropathic pain, chemically-induced pain, and obsessive-compulsive behavior [27,28,36,37,38,39].

In this study, we begin to assess the in vivo antinociceptive efficacy of CMPI, as one of the most selective nAChR PAMs identified. We compare CMPI and dFBr ability to alleviate acute thermal pain in male and female adult Sprague–Dawley rats, using the tail-flick and hot-plate tests. These tests are extensively used to determine the antinociceptive effectiveness of drugs and believed to be mediated by nociceptive responses at spinal and supraspinal levels [40,41]. Our results, shown in Figure 2, established that dFBr is more effective than CMPI in reducing the hot plate test’s acute thermal nociception. Pretreatment with dFBr increased acute thermal pain latency by 53% MPE, whereas CMPI up to 20 mg/kg only increased acute thermal latency by <8% MPE. Unlike CMPI, the effect of dFBr was dose-dependent and statistically significant (P < 0.0001), compared to vehicle pretreatment. The effect of dFBr was also statistically significant (P < 0.0001), when compared to pretreatment with an equal dose of CMPI. However, the tail flick test results, shown in Figure 3, indicate that dFBr is less effective in the tail flick than the hot plate test, whereas dFBr only reduced acute thermal nociception to a similar level as CMPI (15 and 17%, respectively). Despite its direct effect in the hot plate test, dFBr did not enhance the nicotine-induced acute thermal antinociceptive effect in the hot plate or tail flick test results shown in Figure 4. The different effects of dFBr in tail flick versus hot plate tests are not surprising, because different responses in these tests have been previously reported for antinociceptives. For example, Langerman et al. have reported a higher effect of the same dose of morphine in the tail flick than the hot plate test [41]. Dissimilar effects of nAChR PAMs in the tail flick versus hot plate tests could also be attributed to the fact that different sites of the central nervous system mediate the response of these tests. The tail flick and hot plate tests are believed to be mediated via spinal and supraspinal responses, respectively [41].

The α4β2* nAChR activity of dFBr and CMPI could also contribute to the differences observed in the hot plate and tail flick tests. Both dFBr and CMPI potentiate the low agonist sensitivity (α4)3(β2)2 nAChR responses with similar potency (*EC_50_*s ~ 0.3 µM) and efficacy (~ 400% at 1 µM) [31]. Nevertheless, dFBr and CMPI differ in all other pharmacological aspects tested so far, including the ability to potentiate the high agonist sensitivity (α4)2(β2)3 nAChR [33,42], the location of their binding sites in the α4β2* nAChR [32,34,43], effects on potency and efficacy of ACh dose-response curve [31,42], and the ability to penetrate the blood-brain barrier [24,36]. These differences contribute to the higher dFBr effect on acute thermal latency than CMPI observed in the hot plate test. Alternatively, the higher effect of dFBr can be due to differences in the nAChR subtypes involved in thermal nociception. A simple interpretation would be that the (α4)3(β2)2 nAChR contributes differently to spinal and supraspinal thermal nociceptive responses. Studies using α4 or β2 nAChR subunit null mutant (gene knockout) mice have suggested a more prominent role for α4β2* nAChR in nicotine-induced antinociception in the hot-plate test, compared to the tail-flick assay [11]. More elaborate studies are required to examine this possibility. The lack of CMPI effect on the hot plate and tail flick tests was not a result of motor function impairment that is caused by CMPI treatment. The ability of rats to maintain balance on the rotating textured drum of a RotaRod (at 10 rotation per min in IITC life Science model#755) tested 60 min after treatment with 5 or 10 mg/kg CMPI was not different from the vehicle treated group or from pretreatment values (data not shown).

PAMs of nAChRs, alone or added to low doses of an agonist, potentially provide a better alternative strategy to nAChR agonists in replacing opioids for the treatment of pain. They exhibited antinociceptive effects on their own and enhanced analgesic effects without enhancing side effects when coadministered with agonists [15,26]. Here, we have shown that a nAChR PAM with a broader α4-nAChR subtypes spectrum would be more advantageous than a selective (α4)3(β2)2 nAChR PAM in treating acute pain conditions. Still, PAMs acting on the (α4)3(β2)2 nAChR provide a considerable selectivity that potentially limits the side effects, due to binding at other nAChR subtypes. More studies are required to understand the expression and functions of (α4)3(β2)2 nAChR and to examine the merit of (α4)3(β2)2 nAChR-selective PAMs in chronic pain models and other pathologies involving the nAChRs. 

## 4. Materials and Methods

### 4.1. Animals

All animal procedures described in this study were performed using a protocol approved by the institutional animal care and use committee (IACUC) of The Texas A&M Health Science Center-Institute of Biotechnology. Sprague–Dawley rats (150–300g; males and females) were purchased from Envigo and housed at an Assessment and Accreditation of Laboratory Animal Care (AAALAC, Frederick, MD USA)-accredited facility, under standard environmental conditions (food/water ad libitum, 12 h light/dark-light cycle, RT 24 °C).

### 4.2. Drugs

The dFBr, CMPI, and nicotine were purchased from Tocris Bioscience (Minneapolis, MN, USA). Tween 80, propylene glycol, and normal saline (0.9% NaCl) were purchased from Acros Organic (part of Thermo Fisher Scientific, NJ), Amresco (Solon, OH.), and BDHR VWR analytical (Radnor, PA), respectively. For i.p. injection, CMPI and dFBr were dissolved in a vehicle mixture consisting of saline, Tween 80, and propylene glycol, at a ratio of 18:1:1, respectively [38]. For s.c. injection, nicotine was dissolved in physiological saline.

### 4.3. Acute Thermal Nociceptive Tests

Latency to acute thermal nociceptive stimulus in Sprague–Dawley rats was measured using hot-plate and tail flick tests using previously established procedures [44]. Rats were acclimatized to the test room for at least 30 min, and 2-4 control baseline acute thermal pain latencies 15–30 min apart were recorded. At least 15 min after the last baseline recording, rats were treated with an i.p. injection of vehicle or specified doses of nAChR ligands, and the latencies to acute thermal pain reaction were recorded at 15, 30, 60, 90, 120, and 180 min after injection. Vehicle was injected at a dose equal to 1 μl per kg of rat weight, and doses of nAChR ligands were determined as mg per kg of rat weight and dissolved in a volume of vehicle equal to 1 μl per kg of rat body weight. All experiments were performed during the light cycle. Experimenters were blind to treatment used at the time they performed the hot plate and tail flick test. Rats exhibiting signs of locomotor deficiency in the RotaRod test, enhanced thermal pain sensitivity, or abnormal coloration in tail or paws, were excluded from further testing.

#### 4.3.1. Hot Plate Test

Hot plate tests were performed at a temperature of 55 °C using Hot Plate Analgesia Meter (IITC Inc.) Rat reaction time (latency to acute thermal pain in seconds) was recorded as time elapsed after placing on the hot plate metal surface until rat started licking its hind paws. Trials were ended by quick removal of rats from the hot plate surface once a reaction is observed or after a 20-s cut-off time if no reaction was observed. The number of male and female (M/F) rats per treatment group were as follows: dFBr 5 mg/kg (4/4), dFBr 10 mg/kg (4/4), dFBr 20 mg/kg (4/4), CMPI 2 mg/kg (6/6), CMPI 5 mg/kg (6/6), CMPI 10 mg/kg (6/6), CMPI 20 mg/kg (6 males), nicotine 0.5 mg/kg (4/4), nicotine 0.5 mg/kg + dFBr 5 mg/kg (4/4), and nicotine 0.5 mg/kg + dFBr 10 mg/kg (4/4).

#### 4.3.2. Tail Flick Test

The tail flick test was performed using the Tail Flick Analgesia Meter (IITC Inc.) Rats were held in the apparatus with the rat tail positioned under an infrared beam, focused on an area of 4 × 6mm as a heat source. Rat reaction time (latency to acute thermal pain in seconds) was recorded using a built-in sensor, as time elapsed after placing the tail under the infrared beam until the rat sensed stimulation and moved its tail away from the heat source. The trials ended by stopping the heat source once the built-in sensor detected the tail flick or after a 10-s cut-off time if the animal showed no tail flick response. The number of male and female (M/F) rats per treatment group were as follows: dFBr 5 mg/kg (8/8), dFBr 10 mg/kg (8/8), dFBr 20 mg/kg (4/4), CMPI 2 mg/kg (6/6), CMPI 5 mg/kg (6/6), CMPI 10 mg/kg (6/6), CMPI 20 mg/kg (6 males), nicotine 0.5 mg/kg (8/8), nicotine 0.5 mg/kg + dFBr 5 mg/kg (4/4), and nicotine 0.5 mg/kg + dFBr 10 mg/kg (4/6).

### 4.4. Data Analyses

Data analyses were performed using Excel 2010 (Microsoft Corporation) and the GraphPad Prism software, version 8.4.2 (GraphPad Software, Inc., La Jolla, CA). Latencies to acute thermal nociceptive stimulus recorded in hot plate and tail flick tests were converted to the percentage of maximum possible effect (%MPE), using Equations (1) and (2), respectively.

%MPE = [(TL − CL) / (20 − CL) × 100](1)

%MPE = [(TL − CL) / (10 − CL) × 100](2)

where TL is the latency at the specified time after treatment and CL is the baseline control latency recorded before treatment. Data were plotted in figures as mean %MPE ± SD and analyzed for statistical significance using two-way ANOVA with Holm–Sidak’s multiple comparisons test (GraphPad Prism software). Statistical differences (p-values < 0.05) of drug treatment versus vehicle group, when present, are indicated in figures with asterisks as * = *p* < 0.05, ** = *p* < 0.01, *** = *p* < 0.001, and **** = *p* < 0.0001. Significant differences of among drug treatments, when present, are indicated in figures with hashtag symbols as # = *p* < 0.05, ##= *p* < 0.01, ### = *p* < 0.001, ####= *p* < 0.0001.
